# The loss of photosynthesis pathway and genomic locations of the lost plastid genes in a holoparasitic plant *Aeginetia indica*

**DOI:** 10.1186/s12870-020-02415-2

**Published:** 2020-05-08

**Authors:** Jingfang Chen, Runxian Yu, Jinhong Dai, Ying Liu, Renchao Zhou

**Affiliations:** State Key Laboratory of Biocontrol and Guangdong Provincial Key Laboratory of Plant Resources, School of Life Sciences, Sun Yat-sen University, Guangzhou, 510275 China

**Keywords:** *Aeginetia indica*, Plastid genome, Transcriptome

## Abstract

**Background:**

With three origins of holoparasitism, Orobanchaceae provides an ideal system to study the evolution of holoparasitic lifestyle in plants. The evolution of holoparasitism can be revealed by plastid genome degradation and coordinated changes in the nuclear genome, since holoparasitic plants lost the capability of photosynthesis. Among the three clades with holoparasitic plants in Orobanchaceae, only Clade VI has no available plastid genome sequences for holoparasitic plants. In this study, we sequenced the plastome and transcriptome of *Aeginetia indica*, a holoparasitic plant in Clade VI of Orobanchaceae, to study its plastome evolution and the corresponding changes in the nuclear genome as a response of the loss of photosynthetic function.

**Results:**

The plastome of *A. indica* is reduced to 86,212 bp in size, and almost all photosynthesis-related genes were lost. Massive fragments of the lost plastid genes were transferred into the mitochondrial and/or nuclear genomes. These fragments could not be detected in its transcriptomes, suggesting that they were non-functional. Most protein coding genes in the plastome showed the signal of relaxation of purifying selection. Plastome and transcriptome analyses indicated that the photosynthesis pathway is completely lost, and that the porphyrin and chlorophyll metabolism pathway is partially retained, although chlorophyll synthesis is not possible.

**Conclusions:**

Our study suggests the loss of photosynthesis-related functions in *A. indica* in both the nuclear and plastid genomes. The lost plastid genes are transferred into its nuclear and/or mitochondrial genomes, and exist in very small fragments with no expression and are thus non-functional. The *Aeginetia indica* plastome also provides a resource for comparative studies on the repeated evolution of holoparasitism in Orobanchaceae.

## Background

Chloroplast (plastid) is an organelle of plants that conducts photosynthesis, and the structure and gene content of chloroplast genomes are highly conserved in most flowering plants [1]. Typical chloroplast DNA (cpDNA) is circular, ranging mainly from 110 to 160 kb in length [2], and it contains two inverted repeat (IR) sequences separated by a large single-copy region (LSC) and a small single-copy region (SSC) [3]. Holoparasitic plants offer a good system to study plastid genome evolution due to their loss of photosynthetic capacity. They usually display a reduction of plastid genome including genome size and gene content. The family Orobanchaceae is especially suitable for studying chloroplast (plastid) genome evolution because it contains a full trophic spectrum from autotrophic plants, to hemiparasites and holoparasites. In Orobanchaceae, holoparasites occur in three of six well supported clades, namely, Clade III (Orobancheae, ~ 180 holoparasitic species), Clade V (Rhinantheae, 7 holoparasitic species) and Clade VI (Buchnereae, ~ 70 species) [4–6]. So far, plastid genome sequences of holoparasites in Orobanchaceae were mainly from Clade III [7–12]. In Clade V, the plastid genome of only one holoparasite, *Lathraea squamaria*, has been sequenced recently [12]. Although Clade VI includes ~ 70 species from four holoparasitic genera (*Hyobanche*, *Harveya*, *Aeginetia* and *Christisonia*), no plastid genome sequences of holoparasitic plants from this clade have been characterized.

Plastid genomes of holoparasites in Clade III and Clade V of Orobanchaceae differ markedly in genome size and gene content. Plastid genome sizes of holoparasites in Clade III range from 45,673 (*Conopholis americana*) to 120,840 bp (*Orobanche californica*) [9]. However, the plastid genome size of *Lathraea squamaria* from Clade V is 150,504 bp [12], much larger than those in Clade III. The number of intact genes in the plastid genomes of *Conopholis americana* and *Orobanche* species ranges from 21 to 34 [9], and almost all genes related to photosynthesis (*pet*, *psa*, *psb*, and *rbcL*) were lost or became pseudogenes. Whereas in the plastid genome of *Lathraea squamaria*, there are 46 intact genes including many genes related to photosynthesis (such as *psa*, *psb* and *pet*). This might be due to holoparasitic lineages in Clade V is younger than those in Clade III [12].

In addition to plastome degradation, the nuclear genomes of holoparasitic plants are also expected to evolve as a response of the loss of photosynthesis capability, since the genes related to photosynthesis in the plastid genome interact with many genes in the nuclear genome [7, 13]. The expressional changes of nuclear genes could be revealed by transcriptome sequencing. For example, the expression of genes in the photosynthesis and chlorophyll synthesis pathways has been examined in some parasitic plants [7, 14, 15].

*Aeginetia* is a small holoparasitic genus of Orobanchaceae and it consists of about four species distributed in southern and southeastern Asia [16]. According to the phylogenetic analyses of Orobanchaceae, *Aeginetia*, along with *Hyobanche*, *Harveya* and *Christisonia*, forms a monophyletic holoparasitic lineage in Clade VI [4, 5]. *Aeginetia indica* is the most widespread species in this genus [17]. It usually parasitizes on the roots of Poaceae plants like *Miscanthus* and *Saccharum* [18]. In a recent study, transcriptome data of *A. indica* have been used to detect horizontally transferred genes from Fabaceae and Poaceae species [19]. So far, plastid genome sequence and the degradation of photosynthesis related pathways have not been studied in this holoparasitic plant.

In this study, we assembled the plastid genome of *A. indica* using Illumina short reads produced by genome skimming. We also sequenced the transcriptomes from multiple tissues to examine the expressional changes of genes involved in photosynthesis. In addition, we investigated the evolutionary fates of the lost plastid genes in *A. indica*. The results of this study will contribute to our understanding of the coordinated evolution of plastid and nuclear genomes and also facilitate comparative analysis of convergent evolution of holoparasitism in Orobanchaceae.

## Results

### Severe shrinkage of the *A. indica* plastid genome and evolutionary fates of the lost plastid genes

**Fig. 1:**
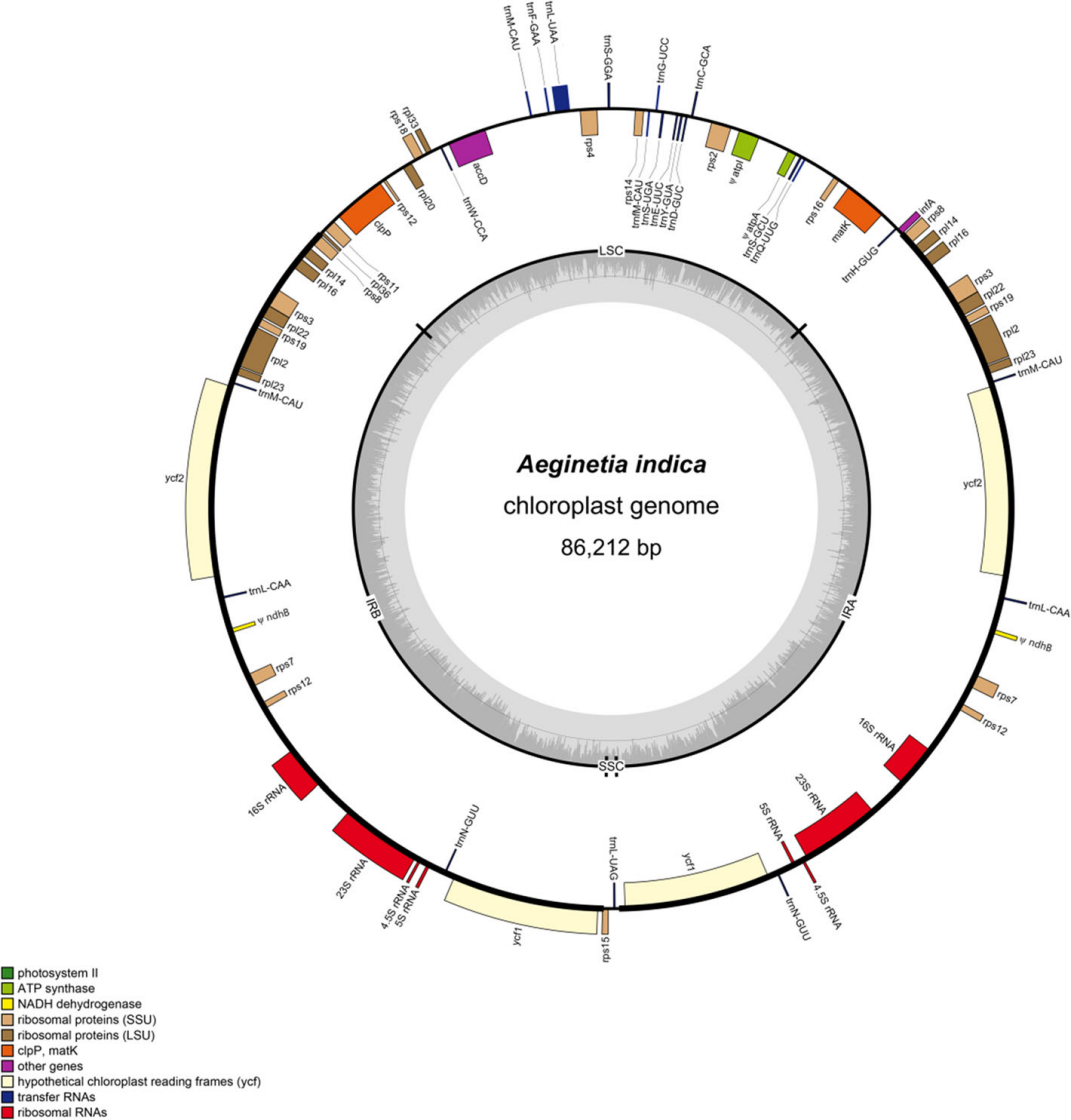
The plastid genome map of *Aeginetia indica*. Genes shown outside and inside the outer circle are transcribed counterclockwise and clockwise, respectively. GC and AT contents across the chloroplast genome are shown with the dark and light shading, respectively, inside the inner circle

The complete plastid genome of *A. indica* is 86,212 bp in length, highly reduced relative to the size of most other angiosperms. It has a typical quadripartite structure, with 22,301 bp of the LSC region, 529 bp of the SSC region, and 31,691 bp each of the IR regions (Fig. 1). AT content of this plastid genome was 65.64%. Based on the DOGMA and GeSeq annotation, the plastid genome of *A. indica* contains 48 putative intact genes and three pseudogenes. These intact genes contain 18 tRNA genes, 4 rRNA genes, 8 *rpl* genes, 12 *rps* genes and 6 other genes, namely, *ycf1*, *ycf2*, *accD*, *matK*, *infA* and *clpP* (Table 1)*.* The three pseudogenes are ψ*atpA*, ψ*atpI* and ψ*ndhB*. ψ*atpA* and ψ*atpI* genes in the LSC region of *A. indica* plastome became pseudogenes because of being truncated at the 88^th^ codon and a premature stop codon at the 32^nd^ codon, respectively. ψ*ndhB* gene in the IR region became a pseudogene due to an internal stop codon at the 53^rd^ codon.

**Table 1:** Summary of genes in the *Aeginetia indica* plastome

Function	Genes
Ribosomal proteins large subunit	*rpl2, rpl14, rpl16, rpl20, rpl22, rpl23, rpl33*, *rpl36*
Ribosomal proteins small subunit	*rps2, rps3, rps4, rps7, rps8, rps11, rps12*, *rps14*, *rps15*, *rps16*, *rps18*, *rps19*
Transfer RNA genes	*trnH-GUG, trnQ-UUG, trnS-GCU, trnC-GCA, trnD-GUC, trnY-GUA, trnE-UUC, trnS-UGA, trnG-GCC, trnM-CAU, trnS-GGA, trnL-UAA, trnF-GAA*, *trnW-CCA, trnL-UAG, trnN-GUU, trnL-CAA, trnfM-CAU*
Ribosomal RNA genes	*rrn4.5, rrn5, rrn16, rrn23*
Other protein-coding genes	*ycf1, ycf2, accD, clpP, matK, infA*
Pseudogenes	*ψndhB, ψatpA, ψatpI*

The SSC region in plastome of *A. indica* shows a severe reduction in size and only two genes, *rpl15* and *trnL-UAG*, were found in this region (Fig. 1). The two IR regions have undergone expansions towards both the LSC and SSC regions. In the chloroplast genomes of the autotropic relative *Lindenbergia philippensis* and other autotropic plants, an intact *ycf1* gene usually spans the IR and SSC regions, and *rps8*, *rpl14*, *rpl16*, *rps3*, *rpl22* and *rps19* genes were in the LSC region. Whereas, in *A. indica* plastome, there is an intact *ycf1* gene in each of the IR regions, and *rps8*, *rpl14*, *rpl16*, *rps3*, *rpl22* and *rps19* genes all shift into the IR regions.

Gene contents in the plastomes of *A. indica*, four holoparasitic species including *Cistanche deserticola*, *Orobanche austrohispanica* and *Epifagus virginiana* from Clade III, and *Lathraea squamaria* from Clade V, and the autotrophic relative *L. philippensis* in Orobanchaceae, were compared (Table S1). Compared with *L. philippensis*, there is substantial loss of genes in the *A. indica* plastid genome. Ten *ndh* (*ndhA*, *ndhC*, *ndhD*, *ndhE*, *ndhF*, *ndhG*, *ndhH*, *ndhI*, *ndhJ* and *ndhK*) genes were lost, and *ndhB* gene became a pseudogene, they encode subunits of NADH-dehydrogenase complex. All five *psa* (*psaA, psaB, psaC*, *psaI* and *psaJ*) and 15 *psb* (*psbA*, *psbB*, *psbC*, *psbD*, *psbE*, *psbF*, *psbH*, *psbI*, *psbJ*, *psbK*, *psbL*, *psbM*, *psbN*, *psbT* and *psbZ*) genes involved in photosystem I and photosystem II, were lost. Also, all six *pet* (*petA*, *petB*, *petE*, *petF*, *petH* and *petI*) genes, which encode cytochrome b6/f complex subunits with function in photosynthetic electron transport, were missing. In addition, four *atp* (*atpB*, *atpE*, *atpF* and *atpH*) genes encoding F-type ATPase subunits, four genes encoding DNA dependent RNA polymerase (*rpoA*, *rpoB*, *rpoC1* and *rpoC2*), and genes encoding envelop membrane protein (*cemA*), large subunit of Rubisco (*rbcL*), haem attachment factor (*ccsA*), and photosystem assembly factors (*ycf3* and *ycf4*) were lost as well. Similar gene loss was found in the plastomes of two holoparasitic species *Orobanche austrohispanica* and *Epifagus virginiana* from Clade III. Although most of these genes were not lost in the plastomes of *Lathraea squamaria* from Clade V and *Cistanche deserticola* from Clade III, many of them became pseudogenes (Table S1).

To study the evolutionary fates of the lost plastid genes in *A. indica*, we identified their genomic positions through extracting genome skimming reads matched with the reference plastomes of 64 plant species and other analyses (see Methods). A total of 339 contigs with length ≥ 150 bp were assembled from the extracted reads, 76 of them were annotated as fragments of plastid genes lost in the *A. indica* plastid genome. These 76 fragments, ranging from 150 to 3086 bp in length, represent 27 lost plastid genes, including four *atp* genes (*atpA*, *atpB*, *atpE* and *atpF*), seven *ndh* genes (*ndhA*, *ndhB*, *ndhD*, *ndhE*, *ndhF*, *ndhH* and *ndhJ*), four *psa* genes (*psaA*, *psaB*, *psaC* and *psaI*), six *psb* genes (*psbA*, *psbB*, *psbC*, *psbD*, *psbE* and *psbJ*), three *rpo* genes (*rpoB*, *rpoC1* and *rpoC2*), and one gene each for *petB*, *rbcL* and *ycf3* (Table S2). In addition, we found that 15 fragments representing nine genes in the *A. indica* plastid genome were also transferred to mitochondrial and/or nuclear genomes and exist in small fragments. Among these genes, seven (*atpA*, *ndhA*, *ndhB*, *ndhF*, *petB*, *rpoB* and *rpl23*) were transferred to both the mitochondrial and nuclear genomes, 11 (*ndhD*, *ndhE*, *ndhH*, *ndhJ*, *rpl14*, *rpl16*, *rpl2*, *rps12*, *rps14*, *rps3* and *rps4*) were transferred to only the mitochondrial genome, and 18 (*atpB*, *atpE*, *atpF*, *psaA*, *psaB*, *psaC*, *psaI*, *psbA*, *psbB*, *psbC*, *psbD*, *psbE*, *psbJ*, *rbcL*, *rpoC1*, *rpoC2*, *ycf3* and *ycf2*) were transferred to only the nuclear genome based on their sequencing depth. All these fragments were not detected in the transcriptomes of multiple tissues, suggesting that they were non-functional.

### Multiple structural rearrangements in the plastid genome of *A. indica* relative to its autotrophic relative

**Fig. 2:**
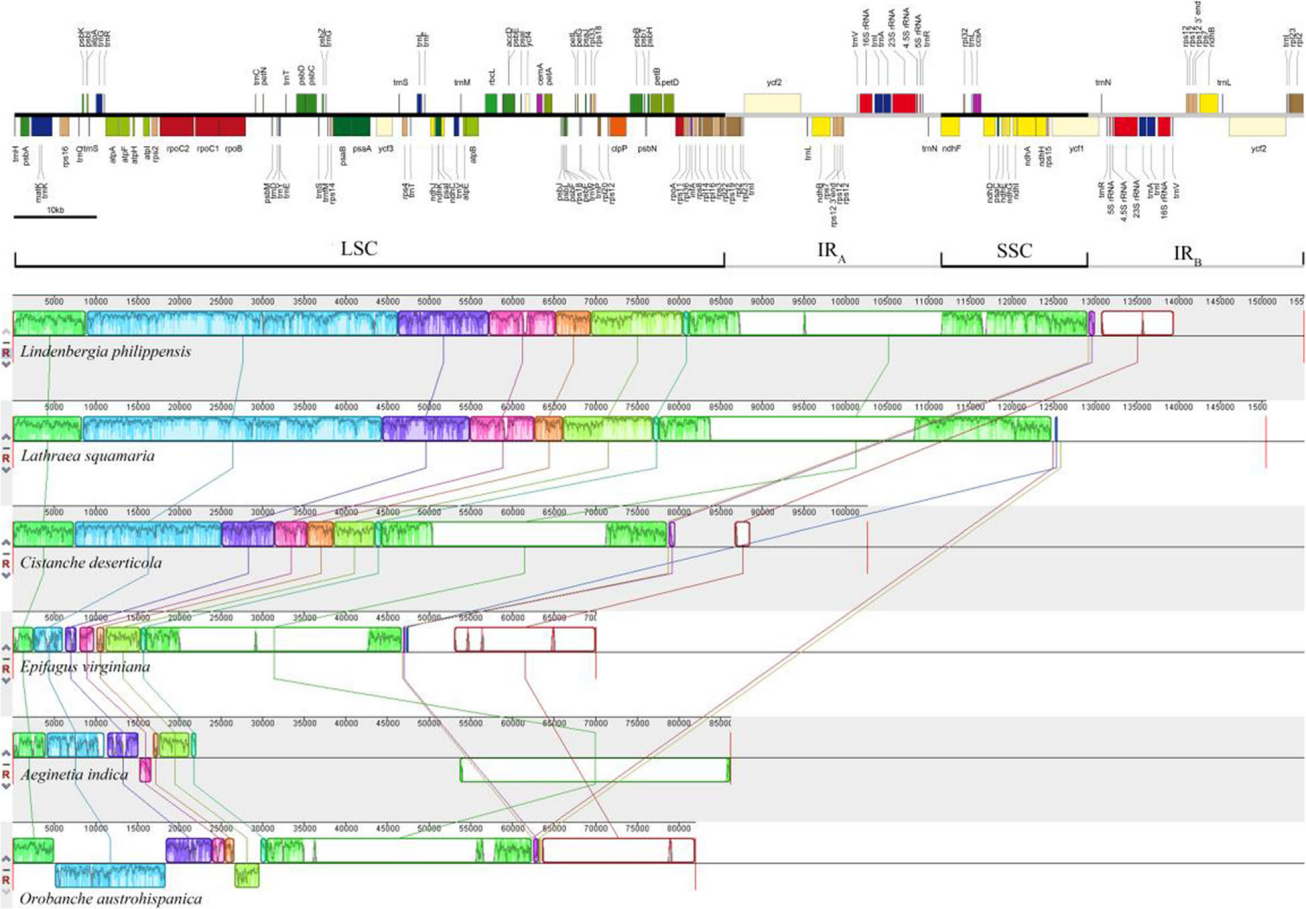
Mauve alignment of the plastomes of *Aeginetia indica* and five other Orobanchaceae species. Gene annotation of the *Lindenbergia philippensis* plastome is the upper panel. Different colors in the alignment show different locally collinear blocks

With Mauve 2.4.0, sequence alignment for the plastomes of *A. indica* (Clade VI) and five other Orobanchaceae species mentioned above was shown in Fig. 2. We identified nine locally co-linear blocks (LCBs) for these six species, and *A. indica* plastid genome has undergone two inversions relative to the chloroplast genome of *L. philippensis*. One inversion is located in the LSC region and contains an intact *accD* gene, and the other contains the intact SSC region and IR_B_ region. Compared with the *L. philippensis* chloroplast genome, there were no inversions in the plastomes of *Lathraea squamaria*, *Cistanche deserticola* and *Epifagus virginiana*, while there were two distinct inversions in that of *Orobanche austrohispanica* (Fig. 2).

### Relaxed purifying selection of *A. indica* plastid genes

A total of 20 protein coding genes shared among the seven species in Orobanchaceae, including 10 *rps* genes, 7 *rpl* genes, and *accD*, *infA* and *matK* genes were used for phylogenetic analysis. The maximum likelihood tree was strongly supported, with bootstrap values of all branches being 100 (Figure S1). Three *Striga* species were clustered into one clade, and *Buchnera americana* was sister to them. *Aeginetia indica* was sister to the clade consisting of the former four species. Non-synonymous (dN)/synonymous (dS) substitution rate ratio (ω) can be considered as an indicator for selection pressure. Two-ratio model (M2) was first compared with one-ratio model (M0). ω values of all genes but *rpl20* and *rps18* in the parasitic plant branch were larger than those of the nonparasitic plant branch (Table S3), and the likelihood ratio test showed that M2 is significantly better than M0 at nine genes, i.e. *accD*, *infA*, *rpl22*, *rps11*, *rps14*, *rps19*, *rps2*, *rps3* and *rps7*, suggesting that these genes were under relaxed purifying selection in parasitic plants. Using three-ratio branch model (M3), we found that hemiparasitic species had higher or much higher ω than holoparasitic species at 13 of 18 genes (ω values of the remaining two genes are not available), while holoparasitic species had slightly higher ω than hemiparasitic species at only five genes (Table S3). This suggests that protein-coding genes retained in the plastome of *A. indica* still play important functional roles rather than experiencing more relaxed selective pressure than hemiparasitic species.

### Degradation of the photosynthesis pathway in *A. indica* revealed by transcriptome analysis

We obtained 21.05, 19.04, 18.34 and 18.02 Gb clean reads for four tissues, i.e. flower, sepal, fruit, and stem, respectively. By de novo assembly of read data from the four tissues, we obtained a total of 205,380 transcripts, among which 153,986 were extracted as unigenes. The average length and N50 of these unigenes were 623.18 and 880 bp, respectively. There were 47,480 ORFs (Open Reading Frames) predicted from all unigenes by TransDecoder, and 42,007 of them could be annotated in Swiss-Prot database, among 42,007 Swiss-Prot annotations, 8466 could be assigned to 131 Kyoto Encyclopedia of Genes and Genomes (KEGG) pathways.

To confirm the reliability of gene expression obtained from transcriptome sequencing, the expression of 10 genes were also examined with qRT-PCR. The expression levels of these genes obtained from the two approaches were of relatively high correlation (Pearson correlation coefficient R^2^ = 0.71; Figure S2), suggesting that gene expression obtained from transcriptome sequencing was reliable.

The photosynthesis pathway (ko00195) from the KEGG pathway database contains 63 genes (30 plastid genes and 33 nuclear genes). In the *A. indica* plastome, genes that encode proteins involved in photosystem I and II, cytochrome *b6f* complex, and photosynthetic electron transport are completely lost. The only two F-type ATPase related genes (*atpA* and *atpI*) in its plastome are pseudogenes. Based on the transcriptome analysis, only 14 nuclear unigenes enriched in the photosynthesis pathway had expression (Table S4). The 14 genes include one gene encoding PSII 6.1 kDa protein, seven genes encoding proteins implicated in photosynthetic electron transport and six genes encoding components of F-type ATPase (Figure S3). Expression of other genes in this pathway was not detected, indicating that these genes were either lost or non-expressional. The results from plastome and transcriptome analyses indicate that the photosynthesis pathway in *A. indica* was completely lost.

The porphyrin and chlorophyll metabolism pathway (ko00860) is complicated in plants. Porphyrins are intermediates of heme and chlorophyll, and heme is required for chlorophyll biosynthesis [20]. In the pathway from glutamate to protoporphyrin IX, the expression of eight genes (*HemA*, *HemB*, *HemC*, *HemD*, *HemE*, *HemF*, *HemL* and *HemY*) were observed in the transcriptome of *A. indica* (Figure S4). However, because of the absence of expression of divinyl chlorophyllide a 8-vinyl-reductase [EC:1.3.1.75], which catalyzes divinyl protochlorophyllide to protochlorophyllide [21], the chlorophyll synthesis pathway appears to end at divinyl-proto-chlorophyllide production in *A. indica* (Figure S4). Obviously, the chlorophyll synthesis pathway is not complete at the later stage and chlorophyll can not be synthesized in *A. indica*.

## Discussion

### Features of the *A. indica* plastid genome

Among the available plastid genomes in Orobanchaceae, the plastid genome of *A. indica* is much smaller than those of *Striga* species (*S. aspera*, *S. forbesii* and *S. hermonthica*) from Clade VI of Orobanchaceae, but similar to those of *Orobanche* species, such as *O. pancicii* (88,525 bp) and *O. crenata* (87,529 bp) from Clade III [10]. However, their plastid genome structures are quite different: the sizes of the LSC (22,301 bp) and SSC (529 bp) regions in *A. indica* are much smaller than those of *O. pancicii* (42,763 and 7608 bp) and *O. crenata* (43,054 and 7925 bp), while the size of IR (31,691 bp) is much larger than those of *O. pancicii* (19,077 bp) and *O. crenata* (18,275 bp). The IR and SSC regions in plastome of *A. indica* have undergone expansions and shrinkage, respectively. These phenomena were also observed in four hemiparasites (*Striga*) from the same clade VI in Orobanchaceae [1]. The IR region in chloroplast genome is usually highly conserved in size and gene content, and it plays an important role in stabilizing chloroplast genome structure [22]. In the present study, IR region expansion occurred in *A. indica* suggests that dramatic changes in the plastome accompanied by the loss of photosynthesis. Moreover, our study shows that holoparasitic plants in Orobanchaceae exhibit different arrangements of plastid genomes, for example, compared with the *L. philippensis* chloroplast genome, there were no inversions in the plastomes of *Lathraea squamaria*, *Cistanche deserticola* and *Epifagus virginiana*, while there were two distinct inversions in each of the *A. indica* and *Orobanche austrohispanica* plastomes.

All 48 genes in the plastome of *A. indica* had sequence length similar to the autotrophic *Lindenbergia philippensis*. The protein-coding genes in *A. indica* play fundamental roles in plastid function, including large and small ribosome protein subunits (*rpl* and *rps* genes), acetyl-CoA carboxylase beta subunit involved in lipid biosynthesis (*accD*), intron splicing (*matK*), translational initiation factor (*infA*), ATP-dependent protease subunit P (*clpP*), and protein import and turnover (*ycf1*) [10, 23]. The function of *ycf2* gene, which has a conserved open reading frame, is still unknown. Four genes, *atpA*, *clpP*, *rpl2* and *rpl23*, contain introns, which is consistent with the retention of *matK*’s function as intron splicing.

The loss of photosynthesis related genes is a common phenomenon in holoparasitic plants, such as *Aphyllon* and *Orobanche* [10, 11]*.* Loss of housekeeping genes was also observed in other holoparasitic plants, for example, the plastid genome of *Balanophora laxiflora* is only 15,505 bp in size, with most genes being lost [24], and *Rafflesia lagascae* has even lost its whole plastid genome [25]. Some housekeeping genes have been transferred to the nuclear genome and their proteins can move back to the plastid to perform their functions [26]. In this study, 27 lost plastid genes in *A. indica* were transferred to nuclear and/or mitochondrial genomes, however, no expressions of these fragments could be detected from our transcriptome data, suggesting that they are nonfunctional.

Previous studies proposed models of plastome evolution in parasites and the order of gene losses [27–29]. The five stages in these models include “Photosynthetic”, “Degradation I”, “Stationary”, “Degradation II” and “Absent” stages. The order of gene losses starts with *ndh* genes, followed by *psa/psb* genes and *rpo* genes, then *atp* genes, *rbcL* gene, nonessential housekeeping genes and other metabolic genes like *accD*, *clpP*, *ycf1* and *ycf2*, ends with the remaining housekeeping genes like *rpl* and *rps* genes. According to their models, plastome of *A. indica* is in the “Stationary” stage.

### The loss of photosynthesis pathway in *A. indica*

*Aeginetia indica* has no photosynthetic activity and obtains all carbon through connection with its host [19]. In the present study, the loss of the photosynthesis pathway in *A. indica* was confirmed based on the loss of photosynthesis genes in its plastome and no detected expression of many genes in the photosynthesis pathway from its transcriptome. Chlorophyll is the primary pigment which absorbs light energy in photosynthesis. Chlorophyll synthesis is impossible in *A. indica* because some key genes in the later stage of the porphyrin and chlorophyll metabolism pathway were not detected with expression. Accumulation of protochlorophyllide in the light may increase the oxidative risk [30], so the degraded chlorophyll synthesis pathway in *A. indica* may benefit for its survival and evolution. In contrast, an intact chlorophyll synthesis pathway was ever found in a holoparasitic plant *Phelipanche aegyptiaca*, suggesting that the expression of the chlorophyll synthesis pathway has other functions (like metabolic signaling, previous studies have proved Mg-protoporphyrin IX is a candidate signaling molecule) other than photosynthesis [31].

## Conclusions

The plastid genome of *Aeginetia indica*, a holoparasitic plant from Clade VI of Orobanchaceae, was de novo assembled in this study. Its plastid genome shows a reduction in size, accompanied with loss or pseudogenization of almost all photosynthesis related genes and some structural rearrangements. The lost plastid genes were transferred into its nuclear and/or mitochondrial genomes, and most of them exist in very small fragments which have no expression and are thus non-functional. Transcriptome analysis from multiple tissues indicates that the photosynthesis pathway of *A. indica* was completely lost, while the porphyrin and chlorophyll metabolism pathway was partially retained, although chlorophyll synthesis is not possible. Our results suggest coordinated loss of photosynthesis related functions in the plastid and nuclear genomes of a holoparasitic plant. The plastome and transcriptome data of *A. indica* in the present study provides genetic resources for future studies and will facilitate comparative analysis of convergent evolution of holoparasitism in Orobanchaceae*.*

## Methods

### Plant materials, DNA isolation and Illumina sequencing

All the tissues of *Aeginetia indica* used in this study were collected from Shimentai Forest Park, Yingde, Guangdong, China. This plant was identified by Dr. Renchao Zhou based on its morphological characteristics described in the Flora Republicae Popularis Sinicae [16]. The voucher specimen (Zhou20190721) was deposited at the herbarium of Sun Yat-sen University (SYS). There were no specific permits required for collecting tissue samples of this species for research purpose. The plants were sampled and frozen immediately in liquid nitrogen, then kept at − 80 °C for further analysis. Total DNA was isolated from the flower stalk with a HiPure Plant DNA Mini Kit (Magen Company, Guangzhou, China) following the manufacturer’s instructions. The quality and quantity of DNA were detected with 1% agarose gel electrophoresis and Qubit 3.0 Fluorometer (Invitrogen Corporation, USA), respectively. Shotgun genome sequencing with paired-end reads of 150 bp was performed on an Illumina Hiseq X Ten platform (IGE Biotechnology Ltd., Guangzhou, China). The sequencing data were deposited in NCBI Sequence Read Archive under accession number SRR9878563.

### Assembly, annotation and alignment of plastid genome

The plastid genome of *A. indica* was de novo assembled from Illumina sequencing data using NOVOPlasty [32], with parameters of insert size (300 bp), K-mer (37) and coverage cut off (1500). The *rps16* gene sequence (EU572719.1) of *A. indica* was served as a seed. Annotation of plastid genome was performed by combining the DOGMA program [33] and GeSeq in MPI-MP CHLOROBOX (https://chlorobox.mpimp-golm.mpg.de/) with default parameters. Genes which contain one or more premature stop codons or frameshift mutations were considered as potential pseudogenes. The annotated plastid genome sequence of *A. indica* was deposited in GenBank under accession number MN529629. The circular map of plastid genome was drawn with OGDRAW [34]. To compare gene loss in the holoparasitic plants from the three clades in Orobanchaceae and potential genomic rearrangements, we downloaded the plastid genome sequences of four holoparasitic species, namely, *Cistanche deserticola* (NC021111.1), *Orobanche austrohispanica* (NC031441.1), *Epifagus virginiana* (NC001568.1) from Clade III, and *Lathraea squamaria* (NC027838.1) from Clade V, and one autotrophic relative *Lindenbergia philippensis* (NC022859.1) from Clade I in Orobanchaceae from GenBank. These sequences were aligned using progressiveMauve [35] with default parameters.

### Identification of genomic locations of the lost plastid genes in *A. indica*

The lost plastid genes in *A. indica* might be transferred to its nuclear and/or mitochondrial genomes. To identify their genomic locations, a total of 64 plastomes from 21 families in four orders (Table S5) were downloaded from GenBank and taken as reference bait sequences. The clean paired-end reads were aligned to all reference bait sequences using Magic-BLAST v1.5.0 [36] with the default parameters. All plastid and plastid-like reads of *A. indica* were extracted by Samtools v1.10 [37] with the command: samtools view -b -S -F 4. All the extracted reads were merged with Samtools v1.10 and then de novo assembled with Velvet v1.2.10 [38] by setting insert length 350, insert length standard deviation 100 and minimal contig length 150. The identity between these contigs and the *A. indica* plastome were obtained using Magic-BLAST v1.5.0. Contigs with identity ≤90% or alignment length shorter than contig length were extracted by Seqtk v1.2-r94 (https://github.com/lh3/seqtk) with the default parameters. All protein sequences of the *Lindenbergia philippensis* chloroplast genome were used to construct a protein database, and the extracted contigs were annotated against this database using BLASTX v2.5.0+ [39] with an e-value ≤10^− 5^. The lost plastid genes were searched from the extracted contigs. Contigs that were annotated as plastid genes or gene fragments with flanking mitochondrial or nuclear sequences were used to determine their genomic positions (mitochondrial or nuclear). However, for most contigs, the sequences are too short to determine their genomic positions, and we therefore calculated the average depth of these contigs by BWA-MEM [40] mapping to infer the genomic locations of the lost plastid genes. This is based on that mitochondrial DNA is usually one to two orders of magnitude in copy number higher than nuclear DNA. Note that for contigs which have hits to the plastid genes of *A. indica*, we calculated the depth of no-hit parts to avoid mapping of plastid gene reads. Sequences of these fragments were searched in the assembled transcriptomes (as described below) to assess whether they are expressional or not.

### Testing signatures of relaxed purifying selection for plastid genes in *A. indica*

We downloaded plastid genomes of six species of Orobanchaceae from GenBank, including four hemiparastic plants *Striga aspera* (MF780872.1), *Striga forbesii* (MF780873.1), *Striga hermonthica* (MF780874.1), and *Buchnera americana* (MF780871.1) from Clade VI, and two autotrophic plants *Lindenbergia philippensis* (NC_022859.1) and *Rehmannia glutinosa* (NC_034308.1). Phylogeny of the six species and *A. indica* was performed using 20 protein-coding genes (7 *rpl* genes, 10 *rps* genes, *accD*, *infA* and *matK*) shared in their plastid genomes. After sequence alignment using MAFFT [41], phylogeny was reconstructed using the maximum likelihood algorithm in RAxML [42] with 1000 bootstrap replicates, with *Rehmannia glutinosa* served as an outgroup. The maximum likelihood tree was shown with FigTree v1.4.3 (http://tree.bio.ed.ac.uk/software/figtree/).

We then used the phylogenetic tree as the input tree to test relaxed purifying selection for plastid genes in *A. indica*. The coding regions of each gene shared among the seven species were aligned by ClustalW (Codons) with default settings in MEGA-X version 10.0.5 [43]. The ratios (ω) of non-synonymous (dN) to synonymous (dS) substitution rate for 20 shared genes were estimated using codon-based analysis (codeml) in the PAML v.4.8a package [44]. Different branch models were used to analyze selective pressures among these species. The null one-ratio model (M0, it hypothesizes that all branches have one ω) was firstly performed, and then the likelihood of a two-ratio model (M2), with a foreground ω1 for parasitic species and a background ω2 for autotrophic species, was compared with that of M0. Moreover, a branch model with three ratios (M3) which assumes three different ω values for holoparasitic, hemiparasitic and autotrophic species, respectively, was compared with M2. The likelihood ratio test for M0 vs M2, and M2 vs M3, was conducted with the Chi-square distribution, with the degree of freedom equal to the difference in the number of parameters for the models, to evaluate the fit of the data to alternative branch models.

### Transcriptome sequencing

Total RNA was isolated from fresh flower, sepal, fruit, and stem tissues of *A. indica*, respectively. The quality and concentration of RNA were determined using 1% agarose gel electrophoresis and a Qubit spectrophotometer, respectively. mRNAs of these four tissues were purified with Oligo dT, and then used to construct cDNA libraries. Four cDNA libraries were sequenced on an Illumina HiSeq2000 platform (IGE Biotechnology Ltd., Guangzhou, China) with PE150 (paired-end 150 bp) strategy. The raw RNA sequencing data of flower, sepal, fruit, and stem tissues of *A. indica* were deposited in NCBI Sequence Read Archive under accession number SRR9959046, SRR9959049, SRR9959048 and SRR9959047, respectively.

### Transcriptome analysis

Raw sequencing data were filtered by removing the adaptors and low quality reads. Transcripts were assembled using clean reads from four tissues through Trinity v2.8.4 [45], and then unigenes were extracted from these transcripts using a perl script get_longest_isoform_seq_per_trinity_gene.pl in Trinity. Expression level of all unigenes (Transcripts Per Million, TPM) was determined using RSEM [46]. For annotation, all unigenes were aligned to NCBI non-redundant database (NR, http://www.ncbi.nlm.nih.gov/genbank/) using DIAMOND (http://ab.inf.uni-tuebingen.de/software/diamond/). Open reading frames (ORFs) were predicted and putative protein sequences were obtained using TransDecoder tools (http://transdecoder.sourceforge.net/). Putative protein sequences were annotated in Swiss-Prot (http://www.uniprot.org/) and Kyoto Encyclopedia of Genes and Genomes (KEGG) [47] pathway (http://www.genome.jp/kegg/pathway.html) databases with the criterion of E value <1e-5 using Blastx and GhostKOALA (https://www.kegg.jp/ghostkoala/), respectively. Within the KEGG pathways, we focused only on the photosynthesis pathway (ko00195) and porphyrin and chlorophyll metabolism pathway (ko00860), and expression of genes in these two pathways were recorded.

### Quantitative real-time PCR experiment

To test the reliability of gene expression obtained from transcriptome sequencing, quantitative real-time PCR (qPCR) for 10 genes was performed using CFX Manager (Bio-Rad, USA) with TB Green® Premix Ex Taq™ II (Tli RNaseH Plus) (Takara Bio Inc., Japan). The protocol of the qPCR was as follows: an initiation step at 95 °C for 2 min, followed by 40 cycles of 5 s at 95 °C, 30 s at 60 °C, and a final melting curve analysis. Each reaction was performed with three biological replicates and three technical replicates, respectively. The *actin* gene was used as the internal control. Primers used for qPCR were listed in Table S6. The expression level for each gene was calculated using the 2^−ΔΔCt^ method [48].

## Supplementary information

**Additional file 1: Figure S1.** Maximum likelihood tree of seven species in Orobanchaceae based on sequences of 20 plastid genes shared among them. Numbers in the nodes are bootstrap values. Scale in substitutions per site.

**Additional file 2: Figure S2.** The correlation between the transcriptome analysis and the qRT-PCR measurements. Each qRT-PCR reaction was performed with three biological replicates and three technical replicates.

**Additional file 3: Figure S3.** The expression of genes in the photosynthesis pathway observed in the *Aeginetia indica* transcriptome. Genes with detected expression were in the red boxes. With courtesy of© www.genome.jp/kegg/kegg1.html.

**Additional file 4: Figure S4.** The expression of genes in the porphyrin and chlorophyll metabolism pathway detected in the *Aeginetia indica* transcriptome. Genes with detected expression were in the red boxes. With courtesy of© www.genome.jp/kegg/kegg1.html.

**Additional file 5: Table S1.** Gene contents of the plastomes of five holoparasitic plants and one autotrophic relative *Lindenbergia philippensisin*. Black boxes indicate the absence of genes, white boxes indicate the presence of genes, and gray boxes indicate pseudogenes.

**Additional file 6: Table S2.** Genomic locations of the lost and transferred plastid genes in *Aeginetia indica*. The annotation, sequence alignment, alignment position and sequence identity are all based on the chloroplast genes of *Lindenbergia philippensis*. * indicates gene also present in the plastome of *Aeginetia indica*.

**Additional file 7: Table S3**. Relaxation of purifying selection in parasitic plants of Orobanchaceae based on branch model analysis of 20 protein coding genes shared by seven species of Orobanchaceae. The likelihood ratio test was used to compare the three models (M0: one ratio model; M2: two ratio model; M3: three ratio model). *P*-values are in bold when they are less than 0.05.

**Additional file 8: Table S4.** Expression level of unigenes of *Aeginetia indica* in the photosynthesis pathway based on transcriptome analysis.

**Additional file 9: Table S5.** Data sources of 64 reference plastid genomes used in this study.

**Additional file 10: Table S6.** Primers used for quantitative real-time PCR.

## Data Availability

The shotgun genome sequencing data of *Aeginetia indica* were deposited in NCBI Sequence Read Archive under accession number SRR9878563; the annotated plastid genome sequence of *A. indica* was deposited in GenBank under accession number MN529629; and the raw RNA sequencing data of flower, sepal, fruit, and stem tissues of *A. indica* were deposited in NCBI Sequence Read Archive under accession number SRR9959046, SRR9959049, SRR9959048 and SRR9959047, respectively. The data sets supporting the results of this study are included in this manuscript and its additional files.

## Abbreviations

cpDNA: Chloroplast DNA
IR: Inverted Repeat
LSC: Large single-copy region
SSC: Small single-copy region
DOGMA: Dual Organellar GenoMe Annotator
LCB: Locally co-linear block
ORF: Open Reading Frame
KEGG: Kyoto Encyclopedia of Genes and Genomes
PSII: Photosystem II
