# Supplementary material for: The loss of photosynthesis pathway and genomic locations of the lost plastid genes in a holoparasitic plant Aeginetia indica
Source: BMC Plant Biol. 2020 May 8;20:199. doi: 10.1186/s12870-020-02415-2 (PMC7206726; doi:10.1186/s12870-020-02415-2)
Supplement: Supplementary file 1 — Additional file 1: Figure S1. Maximum likelihood tree of seven species in Orobanchaceae based on sequences of 20 plastid genes shared among them. Numbers in the nodes are bootstrap values. Scale in substitutions per site. [file 12870_2020_2415_MOESM1_ESM.docx]

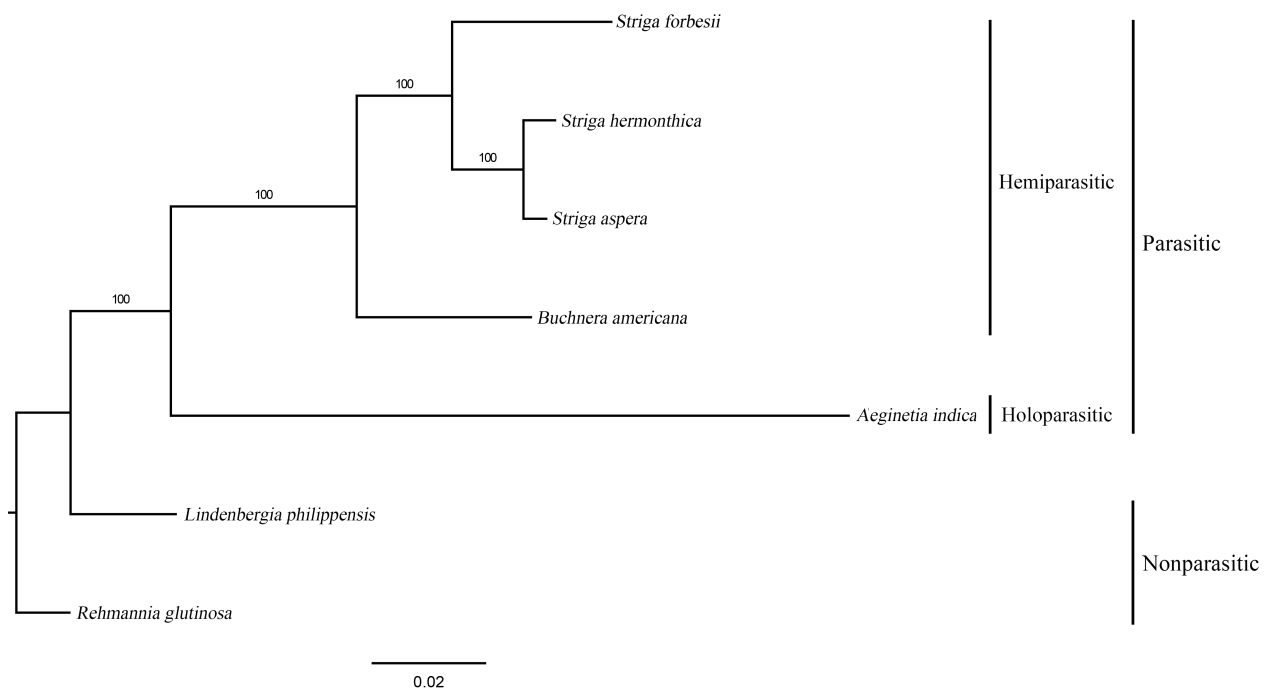


**Figure S1**. Maximum likelihood tree of seven species in Orobanchaceae based on sequences of 20 plastid genes shared among them. Numbers in the nodes are bootstrap values. Scale in substitutions per site.
