# Supplementary material for: The loss of photosynthesis pathway and genomic locations of the lost plastid genes in a holoparasitic plant Aeginetia indica
Source: BMC Plant Biol. 2020 May 8;20:199. doi: 10.1186/s12870-020-02415-2 (PMC7206726; doi:10.1186/s12870-020-02415-2)
Supplement: Supplementary file 2 — Additional file 2: Figure S2. The correlation between the transcriptome analysis and the qRT-PCR measurements. Each qRT-PCR reaction was performed with three biological replicates and three technical replicates. [file 12870_2020_2415_MOESM2_ESM.docx]

**Figure S2.** The correlation between the transcriptome analysis and the qRT-PCR measurements. Each qRT-PCR reaction was performed with three biological replicates and three technical replicates.
