# Supplementary material for: The loss of photosynthesis pathway and genomic locations of the lost plastid genes in a holoparasitic plant Aeginetia indica
Source: BMC Plant Biol. 2020 May 8;20:199. doi: 10.1186/s12870-020-02415-2 (PMC7206726; doi:10.1186/s12870-020-02415-2)
Supplement: Supplementary file 3 — Additional file 3: Figure S3. The expression of genes in the photosynthesis pathway observed in the Aeginetia indica transcriptome. Genes with detected expression were in the red boxes. With courtesy of© www.genome.jp/kegg/kegg1.html. [file 12870_2020_2415_MOESM3_ESM.docx]

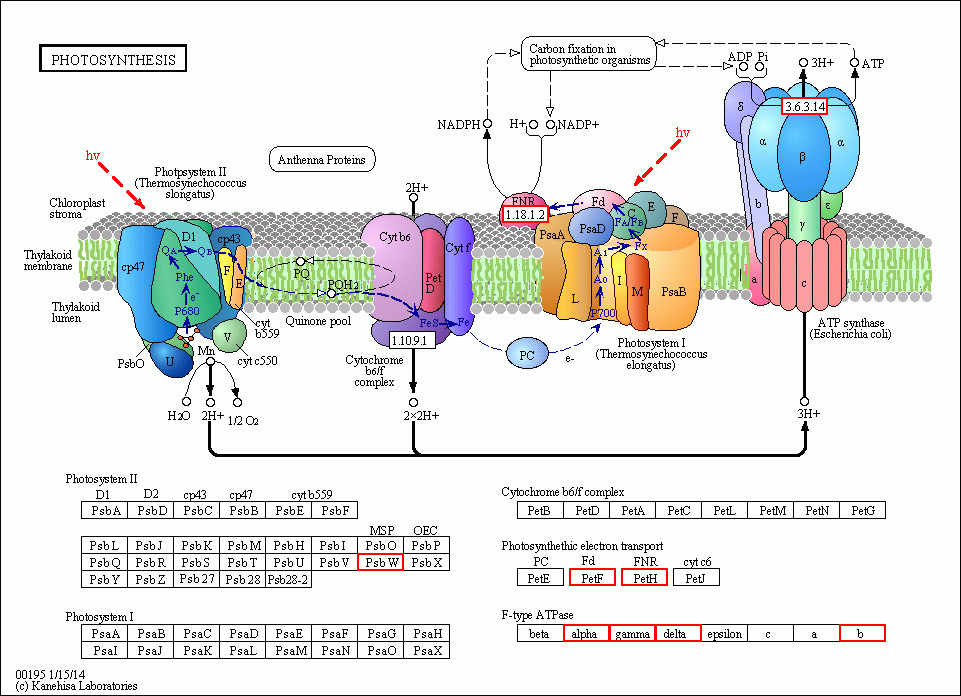


**Figure S3.** The expression of genes in the photosynthesis pathway observed in the *Aeginetia* *indica* transcriptome. Genes with detected expression were in the red boxes. With courtesy of © www.genome.jp/kegg/kegg1.html.
