# Supplementary material for: The loss of photosynthesis pathway and genomic locations of the lost plastid genes in a holoparasitic plant Aeginetia indica
Source: BMC Plant Biol. 2020 May 8;20:199. doi: 10.1186/s12870-020-02415-2 (PMC7206726; doi:10.1186/s12870-020-02415-2)
Supplement: Supplementary file 4 — Additional file 4: Figure S4. The expression of genes in the porphyrin and chlorophyll metabolism pathway detected in the Aeginetia indica transcriptome. Genes with detected expression were in the red boxes. With courtesy of© www.genome.jp/kegg/kegg1.html. [file 12870_2020_2415_MOESM4_ESM.docx]

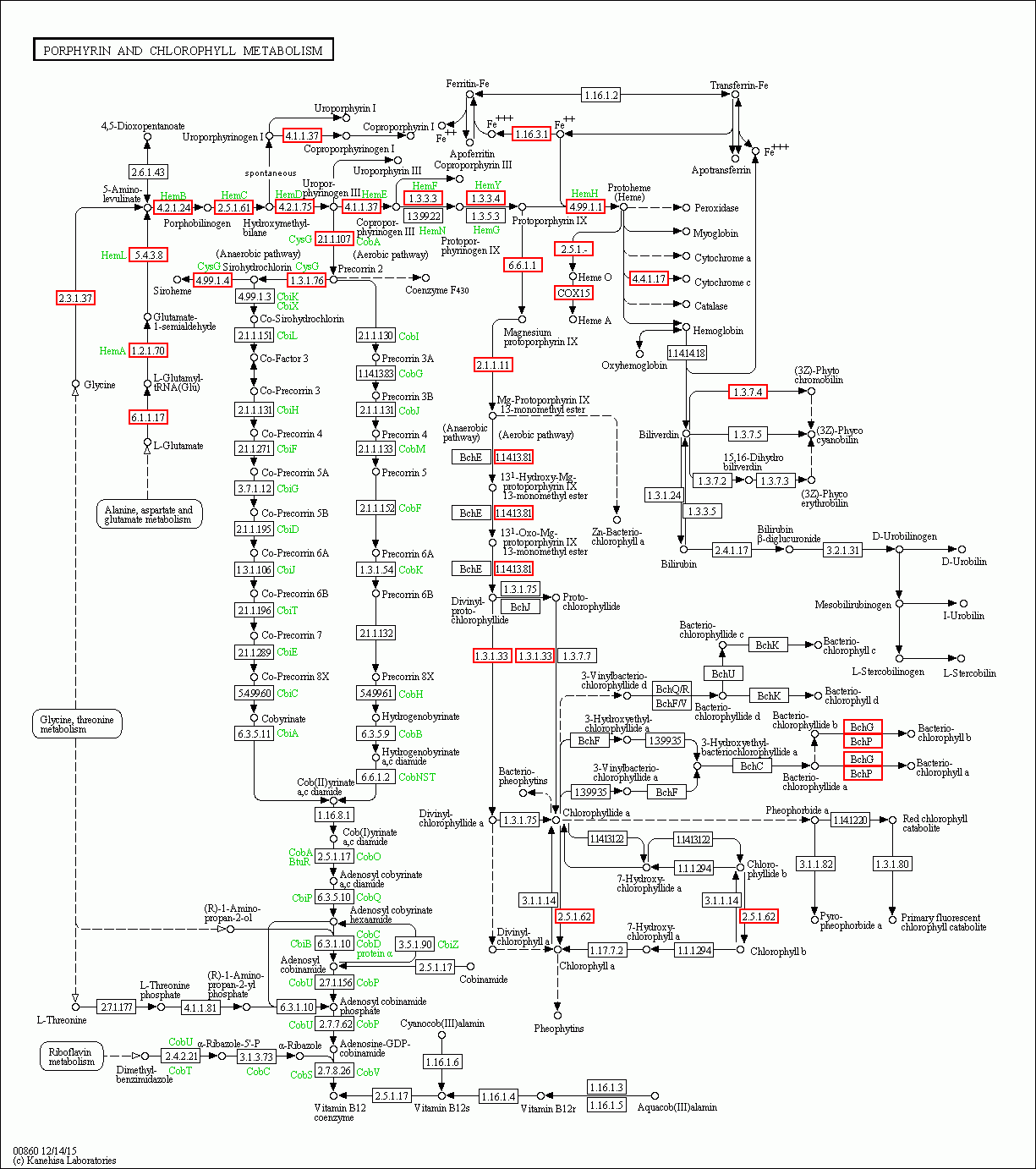


**Figure S4.** The expression of genes in the porphyrin and chlorophyll metabolism pathway detected in the *Aeginetia indica* transcriptome. Genes with detected expression were in the red boxes. With courtesy of © www.genome.jp/kegg/kegg1.html.
