# Supplementary material for: The loss of photosynthesis pathway and genomic locations of the lost plastid genes in a holoparasitic plant Aeginetia indica
Source: BMC Plant Biol. 2020 May 8;20:199. doi: 10.1186/s12870-020-02415-2 (PMC7206726; doi:10.1186/s12870-020-02415-2)
Supplement: Supplementary file 7 — Additional file 7: Table S3. Relaxation of purifying selection in parasitic plants of Orobanchaceae based on branch model analysis of 20 protein coding genes shared by seven species of Orobanchaceae. The likelihood ratio test was used to compare the three models (M0: one ratio model; M2: two ratio model; M3: three ratio model). P-values are in bold when they are less than 0.05. [file 12870_2020_2415_MOESM7_ESM.doc]

**Table S3.** Relaxation of purifying selection in parasitic plants of Orobanchaceae based on branch model analysis of 20 protein coding genes shared by seven species of Orobanchaceae. The likelihood ratio test was used to compare the three models (M0: one ratio model; M2: two ratio model; M3: three ratio model). P-values are in bold when they are less than 0.05.

| **Gene** | **One-ratio model (M0)** |  | **Two-ratio model (M2)** | | |  | **Three-ratio model (M3)** | | | |
| --- | --- | --- | --- | --- | --- | --- | --- | --- | --- | --- |
|  | ω-all |  | ω-nonparasitic | ω-parasitic | P-value（M2-M0） |  | ω-nonparasitic | ω-hemiparasitic | ω-holoparasitic | P-value (M3-M2) |
| *accD* | 0.624 |  | 0.148 | 0.718 | **3.26E-07** |  | 0.16887 | 1.11458 | 0.30019 | **4.60E-10** |
| *infA* | 0.335 |  | 1.00E-04 | 0.40813 | **1.11E-03** |  | 0.0001 | 0.9216 | 0.21733 | **2.22E-03** |
| *matK* | 0.418 |  | 0.412 | 0.42 | 9.38E-01 |  | 0.3986 | 0.39726 | 0.43999 | 6.51E-01 |
| *rpl14* | 0.187 |  | 0.064 | 0.216 | 2.64E-01 |  | 0.11262 | 0.44569 | 0.06939 | 6.08E-02 |
| *rpl2* | 0.448 |  | 0.355 | 0.496 | 5.47E-01 |  | 0.28376 | 0.45951 | 0.84907 | 1.13E-01 |
| *rpl20* | 0.34 |  | 0.467 | 0.32 | 4.98E-01 |  | 0.41993 | 0.2764 | 0.39655 | 5.32E-01 |
| *rpl22* | 0.262 |  | 0.085 | 0.418 | **1.61E-03** |  | 0.17097 | 0.66396 | 0.25294 | 6.21E-02 |
| *rpl23* | 0.992 |  | 0.427 | 1.131 | 4.89E-01 |  | 0.42765 | 0.78018 | 999 | - |
| *rpl33* | 0.307 |  | 0.288 | 0.31 | 9.44E-01 |  | 0.43102 | 0.33219 | 0.25079 | 5.25E-01 |
| *rpl36* | 0.14 |  | 1.00E-04 | 0.186 | 1.07E-01 |  | 0.0001 | 1.3885 | 0.07359 | **9.82E-03** |
| *rps11* | 0.559 |  | 0.181 | 0.688 | **8.06E-03** |  | 0.17001 | 1.27007 | 0.34677 | **7.16E-04** |
| *rps14* | 0.264 |  | 0.029 | 0.356 | **3.36E-03** |  | 0.03563 | 0.71533 | 0.22588 | **6.44E-03** |
| *rps15* | 0.383 |  | 0.177 | 0.452 | 9.30E-02 |  | 0.22215 | 0.49436 | 0.39584 | 4.21E-01 |
| *rps18* | 0.749 |  | 1.072 | 0.729 | 6.30E-01 |  | 1.09573 | 0.85764 | 0.49812 | 2.12E-01 |
| *rps19* | 0.428 |  | 0.045 | 1.039 | **3.12E-04** |  | 0.05341 | 1.55162 | 999 | **-** |
| *rps2* | 0.334 |  | 0.091 | 0.379 | **1.15E-02** |  | 0.10249 | 0.58861 | 0.29465 | **7.55E-03** |
| *rps3* | 0.331 |  | 0.082 | 0.428 | **1.66E-03** |  | 0.09786 | 0.91769 | 0.19848 | **6.02E-05** |
| *rps4* | 0.295 |  | 0.108 | 0.345 | 5.14E-02 |  | 0.09945 | 0.31638 | 0.45541 | 6.41E-02 |
| *rps7* | 0.66 |  | 1.00E-04 | 0.75 | **1.86E-02** |  | 0.11536 | 1.77961 | 0.29525 | **1.40E-02** |
| *rps8* | 0.23 |  | 0.709 | 0.203 | 1.35E-01 |  | 0.42928 | 0.1503 | 0.29629 | 6.04E-01 |

Note: If ω value estimated from codeml are at the upper bound (999), they were recorded as infinity and were not included in further analysis.
