# Supplementary material for: The loss of photosynthesis pathway and genomic locations of the lost plastid genes in a holoparasitic plant Aeginetia indica
Source: BMC Plant Biol. 2020 May 8;20:199. doi: 10.1186/s12870-020-02415-2 (PMC7206726; doi:10.1186/s12870-020-02415-2)
Supplement: Supplementary file 8 — Additional file 8: Table S4. Expression level of unigenes of Aeginetia indica in the photosynthesis pathway based on transcriptome analysis. [file 12870_2020_2415_MOESM8_ESM.docx]

**Table S4**. Expression level of unigenes of *Aeginetia indica* in the photosynthesis pathway based on transcriptome analysis*.*

| **Unigene** | **Annotation** | **Function** | **Length** | **TPM** |
| --- | --- | --- | --- | --- |
| TRINITY_DN777_c0_g1_i1 | PSII 6.1 kDa protein | Photosystem II | 765 | 3.15 |
| TRINITY_DN6763_c0_g1_i1 | Ferredoxin III | Photosynthetic electron transport | 907 | 91.59 |
| TRINITY_DN2565_c0_g1_i2 | Ferredoxin III |  | 954 | 4.08 |
| TRINITY_DN2565_c0_g1_i3 | Ferredoxin III |  | 978 | 9.99 |
| TRINITY_DN469_c1_g1_i1 | Ferredoxin C 2 |  | 837 | 19.36 |
| TRINITY_DN6623_c0_g1_i6 | Ferredoxin III |  | 1162 | 149.82 |
| TRINITY_DN6623_c0_g1_i3 | Ferredoxin III |  | 955 | 17.17 |
| TRINITY_DN2800_c0_g1_i4 | Ferredoxin--NADP reductase, root-type isozyme |  | 1849 | 16.32 |
| TRINITY_DN63111_c0_g1_i1 | ATPase subunit II | F-type ATPase | 792 | 4.74 |
| TRINITY_DN16764_c0_g2_i1 | F-ATPase subunit alpha |  | 3727 | 1.38 |
| TRINITY_DN86782_c0_g1_i1 | F-ATPase gamma subunit 1 |  | 1384 | 4.07 |
| TRINITY_DN76240_c0_g1_i2 | F-ATPase delta chain |  | 1295 | 1.85 |
| TRINITY_DN6616_c0_g2_i1 | Cytochrome c oxidase polypeptide III |  | 6136 | 4.34 |
| TRINITY_DN6616_c0_g2_i2 | Cytochrome c oxidase polypeptide III |  | 3072 | 0.22 |

*TPM = Transcripts Per Million reads
